# Fuzzy-Based Privacy-Preserving Scheme of Low Consumption and High Effectiveness for IoTs: A Repeated Game Model

**DOI:** 10.3390/s22155674

**Published:** 2022-07-29

**Authors:** Laicheng Cao, Min Zhu

**Affiliations:** School of Computer and Communication, Lanzhou University of Technology, Lanzhou 730050, China; zhumin_lut@163.com

**Keywords:** fuzzy trust, repeated game, pollution attacks, camouflage attack, optimal cluster, ETCFS

## Abstract

In the Internet of things (IoTs), data transmission via network coding is highly vulnerable to intra-generation and inter-generation pollution attacks. To mitigate such attacks, some resource-intensive privacy-preserving schemes have been adopted in the previous literature. In order to balance resource consumption and data-privacy-preserving issues, a novel fuzzy-based privacy-preserving scheme is proposed. Our scheme is constructed on a T-S fuzzy trust theory, and network coding data streams are routed in optimal clusters formulated by a designed repeated game model to defend against pollution attacks. In particular, the security of our scheme relies on the hardness of the discrete logarithm. Then, we prove that the designed repeated game model has a subgame-perfect Nash equilibrium, and the model can improve resource utilization efficiency under the condition of data security. Simulation results show that the running time of the proposed privacy-preserving scheme is less than 1 s and the remaining energy is higher than 4 J when the length of packets is greater than 400 and the number of iterations is 100. Therefore, our scheme has higher time and energy efficiency than those of previous studies. In addition, the effective trust cluster formulation scheme (ETCFS) can formulate an optimal cluster more quickly under a kind of camouflage attack.

## 1. Introduction

The Internet of things (IoTs) refers to the networked connection of all daily objects, which can play an eminent role in the application of services based on the Internet of things, such as intelligent fire protection, industrial monitoring, intelligence collection, renewable energy adaptation, and so on, greatly simplifying and bringing convenience to life [1,2]. However, IoT is vulnerable to various network attacks, which can destroy the process of data transmission and increase energy consumption. Therefore, in the previous literature, many privacy-preserving schemes have been proposed to protect the security of data. Furthermore, network coding technology has been introduced into IoTs for protecting the privacy of data, where the sensor data is divided into multiple generations. Specifically, the multiple packets in any generation are signed by an identifier. With this kind of packet mixing, characteristic of the network coding, an internal or external enemy can inject some fake or modified packets into the information flow, making it more vulnerable to contamination attack, so that IoT devices cannot identify the correct and trusted data. In addition, the polluted data will spread widely. In response to network-coding-enabled IoT attack scenarios, we consider two typical types of pollution attacks. We firstly consider intra-generation attacks, where the attacker modifies the innocent packets in multiple generations. Secondly, we consider inter-generation attacks, where the attacker forges the malicious packets into valid packets in one generation. In contrast with previous defense works, we introduce a T-S fuzzy trust evaluation model to defend against malicious IoT devices and construct an energy-efficient privacy-preserving framework. In the T-S fuzzy trust evaluation model, limited bandwidth and power consumption are considered. Meanwhile, our T-S fuzzy trust model can obtain a more accurate trust evaluation value under the premise of ensuring the required stability of IoTs. Referring to [3,4,5], we design a repeated game model to perfect the energy-efficient privacy-preserving scheme, where the subgame Nash equilibrium of the repeated game model can balance the data security and network resource consumption [6]. The contributions of this research are given as follows:

- Firstly, we propose a novel privacy-preserving scheme based on T-S fuzzy trust theory to mitigate the pollution attacks, in which the security is proved according to the hardness of the discrete logarithm.
- Secondly, we construct a repeated game model to formulate the optimal cluster, in which subgame-perfect Nash equilibrium is achieved, and the energy efficiency is higher than in previous research under a kind of camouflage attack.
- Finally, we prove the correctness of our privacy-preserving scheme through strict mathematical derivation and verify the performance superiority of our scheme by simulation.

The organization of this paper is as follows. In Section 2, we present the previous theories, including privacy-preserving schemes, T-S fuzzy technology, and the game theory on which this research is based. In addition, we present a variety of improved models adapted to coding trust in IoTs and discuss the shortcomings of these works in Section 3. After that, we propose the energy-efficient privacy-preserving scheme based on the T-S fuzzy trust model and repeated game model in Section 4. Then, the simulation results and discussion are provided in Section 5, proving the correctness and accuracy of our proposed model. Finally, we draw our conclusions in Section 6.

## 2. Related Works

### 2.1. Privacy-Preserving Schemes

At present, the technologies to solve pollution attacks in network coding can be roughly divided into two categories: information theory schemes and cryptography-based schemes. The information theory scheme mainly prevents pollution attacks by detecting and correcting the polluted packets on the sink node. Regarding the effectiveness of information theory methods, they cannot make intermediate nodes filter out fake messages, which means that they can only passively tolerate the pollution attacks of sink nodes.

Another solution to the problem of pollution attacks is password-based authentication technology that enables transponders to verify the accuracy of packets they receive in routing. This method enables intermediate nodes to detect and discard fraudulent packets in transmission, which can effectively reduce pollution attacks from the source [7].

In [8], a homomorphic signature scheme based on the hardness of the discrete logarithm problem was proposed, which allows a node to check the validity of a packet without decoding it. In this scheme, the node can check the integrity of the received packet by taking advantage of the linearity of the packet in the coding system. In addition, Zhang et al. [9] proposed a new idea called “orthogonal fill” in network coding, combining a signature scheme based on public keys with a MAC scheme based on symmetric keys. This scheme requires updating the public-private key tuple, which results in a high cost of forwarder calculation. Liu and Wang in [10] divided pollution attacks in actual network coding into intra-generation pollution attacks and inter-generation pollution attacks. Each packet of each generation depends on the correct identifier and the corresponding dynamic public key designed for this generation for validation. The scheme works by shuffling static keys, and each shuffling is only used in one generation. However, the scheme only focuses on the prevention of pollution attacks in general.

Besides the authentication scheme based on the asymmetric key, there is also a class of authentication schemes based on symmetric key encryption. To solve the problem of label contamination, Li et al. in [11] proposed a time-based authentication scheme called RIPPLE, which uses delayed MAC key disclosure to achieve security similar to public key authentication schemes. It is the first scheme to consider tag contamination, allowing nodes to effectively detect corrupted packets and encode only validated packets. Cheng and Jiang proposed a homomorphic message authentication code scheme for network coding in [12], which they claimed could obtain a reliable security parameter.

Although the authentication scheme based on the symmetric key has lower computational complexity than that based on the asymmetric key, it still has large bandwidth overhead and key management problems. In [13], Cheng et al. proposed two improved key distribution schemes for, respectively, signature schemes based on homomorphic subspace and label-encoding schemes based on key pre-allocation, which can reduce the homomorphism of messages belonging to two different generations to combat multi-generation pollution attacks; however, their communication costs increase significantly. In [14], Li et al. proposed a multi-source homomorphic network coding signature in the standard model to deal with multi-source devices in an IoTs network system, to ensure network availability while mitigating pollution attacks. In [15], Fiandrotti et al. propose a simple and effective method to deal with contamination attacks in point-to-point flow based on network coding. The scheme can reduce the impact of pollution attacks by selectively combining the packets of the forwarder and proving that the probability of the packet being drawn increases with time. Subsequently, in [16], Antonopoulos et al. introduced a cooperative nonparametric statistical framework to identify and mitigate node misconduct in IoT coding scenarios. The framework does not require monitoring of wireless channels and additional overhead, but it is not resistant to eavesdropping attacks. In [17], Lawrence et al. proposed a scheme based on homomorphic message authentication coding in IoTs that could identify contamination attacks and attack initiating nodes and developed data/message and marker error correction techniques. In addition, Sodhro et al. included cognitive/brainwaves via electroencephalogram (EEG), which function as a unique performance indicator to construct an energy-efficient cognitive authentication scheme [18] for smart healthcare applications, promoting the development of biometric recognition.

### 2.2. T-S Fuzzy

Nonlinearity is a common feature of many real systems [19,20]. It is also an important factor that directly leads to the complexity of system analysis and design. Fortunately, the “universal approval” of the Takagi–Sugeno (T-S) fuzzy model can solve the problems caused by nonlinearity well. Therefore, in the past few years, many studies have modeled nonlinear systems as T-S fuzzy systems, which are locally linear time-invariant systems connected by if-then rules. Consequently, studies on T-S fuzzy systems have attracted more and more attention [21]. Various meaningful studies on T-S fuzzy systems have been carried out. To avoid the deterioration of system performance, fault-tolerant control (FTC) and fault detection and isolation (FDI) schemes based on the T-S fuzzy model are developed in [22]. By using the set theory description of the T-S fuzzy model, aiming at the problem of fault isolation of the T-S fuzzy system, a new fault isolation method was proposed in [23], which does not introduce the measurement information of the fault isolation sensor into the premise variables of the corresponding observer. A new descriptor fuzzy sliding-mode observer approach was proposed in [24], which augments the original fuzzy plant into a descriptor system to estimate the system state, sensor fault, and actuator fault vectors at the same time.

In [25], the problem of the hybrid-triggered controller design with quantization was investigated for a T–S fuzzy system under cyber-attacks. However, in practical applications, parameter uncertainties in membership functions are usually inevitable. This has encouraged research on the sliding mode control problem of interval type-2 (IT2) fuzzy systems subject to the unmeasurable state and cyber-attacks by introducing two weighting factors [26]. In [27], by designing a fault detection observer and separating the measured premise variables explicitly from the unmeasurable ones, the finite frequency error detection problem of T-S fuzzy systems with some unmeasurable premise variables was studied.

### 2.3. Game Theory

The methods of using game theory to mitigate different threats to the security of the Internet of things are broadly summarized, and these methods are classified into cooperative games and non-cooperative games [28]. In addition, some potential research trends with great promising prospects in game theory have been proposed. In [29], a privacy protection solution in an intelligent transportation environment was presented based on a game model consisting of two participants (data holder and data requester). Markov chains were utilized to model transformations for finding the optimal protection strategy for data holders to keep data private over a series of interactions with the data requester. Furthermore, the characteristics of the Stackelberg game are used to model security in IoT applications [30]. At the same time, the Stackelberg game has also been extended to deal with false injected data of intelligent attacks in sensor networks to enhance data trustworthiness [31]. A repeated game model was also presented to enhance the resistance of the Internet of things to selective forwarding attacks [32]. More specifically, in this game, the credibility of high-priority data was maximized by detecting malicious nodes that discard high-priority packets. However, the model attaches too much importance to high-priority packets, which leads to the rapid degradation of low-priority packets due to the impact of unprocessed attacks. Then, cooperative game theory was used to improve security and manage cost and delay, focusing on the trust evaluation process based on mixed-strategy Nash equilibrium [33]. In [29], a repeated game model was proposed to detect and mitigate the influence of malicious cluster members, and a TDMA protocol was adopted to keep the synchronization of cluster heads and cluster members, to reduce the complexity of the detection mechanism.

## 3. System Model

### 3.1. Network Model

In this paper, a linear network coding enabling IoT is considered, in which an IoT device sends a batch of sequenced messages to multiple target nodes. The delivered messages are divided into *M* generations, where each message can be regarded as an *n*-dimensional vector over the finite field $F_{p}$. Here, *p* is a pre-determined prime integer. Meanwhile, each generation contains *m* native messages. Without loss of generality, the *i*-th generation is labeled by an ρ-bit binary string $Id_{i}\in {\left\{0,1\right\}}^{\rho }$, where i∈{1,…,M} and $\rho \geq \left\lceil \log_{2}^{M}\right\rceil$. Let $\Gamma =\{Id_{1},\ldots ,Id_{M}\}$ represent the set of generation identifiers. Then, the set of native messages belonging to the *i*-th generation is defined as $\left\{D_{i,1},\ldots ,D_{i,m}\right\}$, where

(1) $$D_{i,j}=\left(D_{i,j}^{\left(1\right)},\ldots ,D_{i,j}^{\left(n\right)}\right)\in F_{p}^{n},j\in \left\{1,\ldots ,m\right\}$$

In this network model, the trust T between IoT devices is considered. The trusted routing device set in the next round of data transmission is selected by the trust value generated in the previous round of data transmission.

#### 3.1.1. Trust Encoding at Data

For the *j*-th native messages $D_{i,j}$ in the *i*-th generation, a *t*-dimensional unit vector $p_{j}$, with the *j*-th entry being the measurable trustworthiness $T_{i,j}$ for IoT devices and the other being 0, is appended into the native messages. Then, the corresponding augmented block $c_{i,j}$ is given as follows:

(2) $$c_{i,j}=\left(p_{j},D_{i,j}\right)=\left(\overset{t}{\overset{︷}{{\underset{︸}{0,\ldots ,0}}_{j-1},T_{i,j},{\underset{︸}{0,\ldots ,0}}_{t-j}}},D_{i,j}\right)\in F_{p}^{t+n},j\in \{1,\ldots ,t\},$$

according to the bi-linear map polynomial-time algorithm, the corresponding encrypted block is given by

(3) $$\begin{array}{cc} E_{i,j} & =Encrypt(h,Id_{i},c_{i,j})\\ \; & =(\overset{t}{\overset{︷}{{\underset{︸}{0,\ldots ,0}}_{j-1},e_{T_{i,j}},{\underset{︸}{0,\ldots ,0}}_{t-j}}},D_{i,j}), \end{array}$$

where *h* is the parameter in the bi-liner map between two multiplicative cyclic groups, and $Id_{i}$ is the number of IoT devices.

#### 3.1.2. Trust Decoding for Receivers

When the network controller receives the encoding data, the data block is first decrypted and stored in the buffer. After receiving *m* non-linearly correlated data blocks, the network controller can recover the native messages by Gaussian elimination. Then, an ACK message will be fed back to the sender to confirm the transmission of the next generation of messages.

### 3.2. Adversary Model

We assume that there exists an attacker attempting to launch attacks in this network. The types of attacks are listed as follows:

- **Pollution attack:** Attackers attempt to launch malicious data injection attacks to disrupt the data transmission. Then, data integrity and privacy are compromised.
- **Camouflage attack**: Attackers deceive their surrounding trust evaluation devices by pretending to be the normal devices, which leads to the wrong trust measurement results.

### 3.3. T-S Fuzzy Trust Model

Here, the data-privacy-preserving model between IoT devices is introduced. However, we should also consider routing security issues in data transmission. With the development of trust evaluation technology in routing security, Li et al. [34] studied the trust routing model instead of the traditional cryptographic scheme to defend against malicious nodes in IoTs. In practical applications of IoTs, the degree of trustworthiness between IoT devices is usually complex and variable. In this section, we reasonably assume a T-S fuzzy model to mitigate the influence of subjective factors in trust evaluation. The T-S fuzzy model is defined as follows:

**Definition** **1.** *Suppose that the domain $X=\{x_{1},x_{2},\ldots ,x_{n}\}$ is a non-empty set, and $x_{i}\left(i=1,2,\ldots ,n\right)$ is an element in X. For $\forall x_{i}\in X$, there is a mapping relation as follows: $\mu_{T}:X\to \left[0,1\right],x_{i}\mapsto \mu_{T}\left(x_{i}\right)\in \left[0,1\right]$; then, the set $T=\{(x_{1}\left|\mu_{T}\left(x_{1}\right)),\right.(x_{2}\left|\mu_{T}\left(x_{2}\right)),\ldots \right.,\left(x_{n}\left|\mu_{T}\left(x_{n}\right))\right.\right\}$ is defined as a fuzzy subset $\left(\forall x_{i}\in X\right)$ on $X_{\mu_{T}\left(x_{i}\right)}$, which is called the membership degree of $x_{i}$ to fuzzy subset T, and the mapping $\mu_{T}$ is called the membership function of fuzzy subset T.*

In Definition 1, $X=\{x_{1},x_{2},\ldots ,x_{n}\}$ is the set of IoT devices. Here, we chose the communication trust $T_{c}$ and energy trust $T_{e}$ as the fuzzy characters $z_{k}$ to objectively describe the trustworthiness of IoT devices. Therefore, the vector $v\left(x_{ji}\right)=v_{ji}=\left(\mu_{1i},\mu_{2i},\ldots ,\mu_{mi}\right)$ formed by the membership degree of each subject competing for these finite fuzzy parameters $z_{k}$ is used as the evaluation trust vector of $\mu_{ji}\in \left[0,1\right],\left(j=1,2,\ldots ,l\right)$ for $x_{i}$, while $v_{ji}$ is the evaluation trust vector of node j to node i, and $\mu_{ki}\left(k=1,2,\ldots ,m\right)$ is the membership degree of node $i(x_{i})$ to fuzzy parameter $z_{k}$ evaluated by node j. Then, the definition of the fuzzy rule is given as follows:

**Definition** **2.** *IF $v_{1i}$ is $X_{\mu_{T}\left(1,x_{i}\right)}$ and $v_{2i}$ is $X_{\mu_{T}\left(2,x_{i}\right)},\ldots ,v_{ji}$ is $X_{\mu_{T}\left(j,x_{i}\right)}$, THEN*

(4) $$\begin{array}{cc} \; & \dot{x}\left(t\right)=A_{i_{1}i_{2}\ldots i_{p}}x\left(t\right)+B_{i_{1}i_{2}\ldots i_{p}}\left(\mu \left(t\right)+a_{1}\left(t\right)\right)+B_{w}n\left(t\right)\\ \; & y^{j_{1}}\left(t\right)=C^{j_{2}}x\left(t\right),j_{1}=1,\ldots ,m-h\\ \; & y^{j_{2}}\left(t\right)=C^{j_{2}}x\left(t\right)+a_{2}^{j_{2}}\left(t\right),j_{2}=m-h+1,\ldots ,m, \end{array}$$

Where $x\left(t\right)\in R^{n}$ is the network statement, $\mu \left(t\right)\in R^{l}$ denotes the map input, n(t) is the bias of noise, $a_{1}\left(t\right)\in R^{l}$ is the attack intensity in network, $a_{2}\left(t\right)\in R^{l}$ is the transmission bias for indirect trust evaluation, and $y^{j_{1}}\left(t\right)\in R$ and $y^{j_{2}}\left(t\right)\in R$ are respectively the output of direct and indirect trustworthiness in the T-S fuzzy model. In addition, *m* is the number of IoT devices within two hops of node $x_{i}$, and *h* is the number of IoT devices that can communicate directly with node $x_{i}$. Then, *C* is the measurable trustworthiness including communication trust $C_{1}$ and energy trust $C_{2}$, while $A_{i_{1}i_{2}\ldots i_{p}}$, $B_{i_{1}i_{2}\ldots i_{p}}$, and $B_{w}$ are known matrices with suitable dimensions. Then, the y(t) and *C* can be rewritten as follows,

(5) $$y\left(t\right)=\left[\begin{array}{c} y_{1}\left(t\right)\\ y_{2}\left(t\right) \end{array}\right],C=\left[\begin{array}{c} C_{1}\\ C_{2} \end{array}\right],$$

Then, a singleton fuzzifier inference method with center average defuzzifiers is applied to rewrite the T-S fuzzy model as follows:

(6) $$\begin{array}{cc} \dot{x}\left(t\right)= & \frac{1}{\sum_{i_{1}=1}^{r_{1}}\sum_{i_{2}=1}^{r_{2}}\ldots \sum_{i_{p}=1}^{r_{p}}\left(\prod_{j=1}^{p}X_{\mu_{T}\left(1,x_{i}\right)}\right)}\\ \; & \times \sum_{i_{1}=1}^{r_{1}}\sum_{i_{2}=1}^{r_{2}}\ldots \sum_{i_{p}=1}^{r_{p}}\left(\prod_{j=1}^{p}X_{\mu_{T}\left(1,x_{i}\right)}\right)\\ \; & \times \left(A_{i_{1\to p}}x\left(t\right)+B_{i_{1\to p}}\left(\mu \left(t\right)+a_{1}\left(t\right)\right)+B_{w}n\right)\\ y(t)= & Cx\left(t\right)+a_{2}\left(t\right). \end{array}$$

Therefore, we can obtain the objective T-S fuzzy set T={y(1),y(2),…,y(t)},1≤t≤n.

## 4. The Energy-Efficient Privacy-Preserving Scheme Based on T-S Fuzzy Trust Model and Repeated Game Model

**Figure 1:**
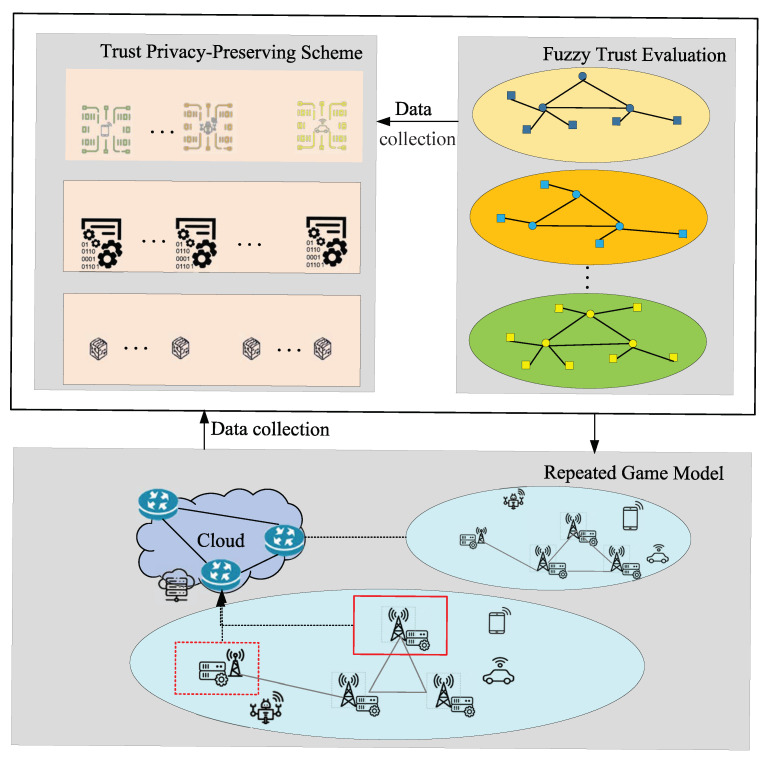
The framework of the fuzzy-based privacy-preserving scheme based on the repeated game. The devices in the solid line frame are common IoT devices in the cluster, and the dotted line is the routing device that guarantees data uploading.

In this section, we introduce the framework of our privacy-preserving scheme. Figure 1 shows the relationship between fuzzy trust evaluation, the repeated game model, and the trust privacy-preserving scheme, in which the repeated game helps the network controller formulate the optimal cluster to send the data to the trust privacy-preserving scheme. The game model can obtain a balance between network performance and resource consumption so that we can ensure maximum network performance by consuming fewer resources. Here, network performance indicators include defense attack capability, energy consumption, and so on. Explanations of this can be found in [35]. Therefore, the IoT data can be safely transmitted with low energy consumption.

### 4.1. A Privacy-Preserving Scheme Based on T-S Fuzzy Trust Model

In this subsection, we propose a privacy-preserving scheme based on the T-S fuzzy model, which can protect data privacy against pollution attacks in coding IoT networks. Firstly, the scheme can be formulated as four steps (**Encrypt**, **Sign**, **Verify**, **Decrypt**). The details of those steps are given as follows:

- **Encrypt** (*h*, T, $Id_{i}$ c). According to Definition 1, the trust set T contains 0 and 1. When the trustworthiness of IoT devices is 1, the coding data will be received. Then, the source is generated as a series of *t*-bit binary strings ${\left\{s_{j}\right\}}_{j=1}^{t}$. A keyed pseudo-random function $f:{\left\{0,1\right\}}^{*}\times {\left\{0,1\right\}}^{*}\times K\mapsto F_{p}$ is applied to generate the encryption matrix
  (7) $$E_{c,T}=\left[\begin{array}{ccc} c_{e_{i,1}} & \; & \\ \; & \ddots & \\ \; & \; & c_{e_{i,t}} \end{array}\right].$$
  Therefore, we rewrite Equation (3) as follows,
  (8) $$\begin{array}{cc} E_{i,j} & =Encrypt(h,T,Id_{i},c_{i,j})\\ \; & =(E_{c,T},D_{i,j}). \end{array}$$
- **Sign** (sk, $Id_{i}$, c). Suppose a full-domain hash function $H:{\left\{0,1\right\}}^{*}\mapsto F_{p}$ as a random oracle. The signature of source *c* is given by
  (9) $$\Delta =\zeta^{\sum_{i=1}^{t+n}c_{i}sk_{i}+\left(\sum_{i=1}^{t}c_{i}\right)H\left(Id_{i}\right)sk_{t+n+1}},$$
  where sk is the signature key such that $sk=\left\{sk_{1},\ldots ,sk_{t+n+1}\right\},sk_{i}\overset{R}{\leftarrow }F_{p}$. Then, the data blocks ${\left\{c_{i}\right\}}_{i-1}^{\sigma }$ and ${\left\{\Delta_{i}\right\}}_{i-1}^{\sigma }$ of the *i*-th generation are combined as follows:
  (10) $$\Theta_{i}=\left(\begin{array}{c} \sum_{i=1}^{\sigma }T_{i}c_{i},\prod_{i=1}^{\sigma }\Delta^{T_{i}},Id_{i} \end{array}\right).$$
- **Verify** (pk, c, $Id_{i}$, Δ). When the public key pk, a data block c, a generation $Id_{i}$, and the signature Δ are given, the compared computation is given by
  (11) $$\eta_{1}=e\left(\Delta ,o\right)$$
  and
  (12) $$\eta_{2}=e\left(\zeta ,\prod_{i=1}^{t+n}h_{i}^{c_{i}}\cdot \prod_{i=1}^{t}h_{t+n+1}^{H\left(Id_{i}\right)c_{i}}\right).$$
  where *o* is the generator of G, $pk=(\zeta ,o,G,G_{D},h)$, and $\mu \overset{R}{\leftarrow }G\left\{1\right\}$. G and $G_{D}$ are two multiplicative cyclic groups, which satisfy $e:G\times G⟼G_{D}$ in a bilinear map, and $h:=\left\{o^{sk_{1}},\ldots ,o^{sk_{t+n+1}}\right\}$. When $\eta_{1}=\eta_{2}$, the verification is successful; otherwise, it fails.
- **Decrypt** (*h*, T, $Id_{i}$ c). When the secret key *k* and the pseudo-random function *f* are given, the decryption matrix can be computed as follows:
  (13) $${\mathrm{DE}}_{c,T}=\left[\begin{array}{ccc} c_{e_{i,1}}^{-1} & \; & \\ \; & \ddots & \\ \; & \; & c_{e_{i,t}}^{1} \end{array}\right]$$

### 4.2. The Correctness and Security Analysis of Our Privacy-Preserving Sheme

In this subsection, we provide the correctness analysis of our privacy-preserving scheme with two theorems and proofs.

**Theorem** **1.** *Given an augmented data block $c\in \prod {}_{i}$ including coding vector p and native message D, $\mathrm{Decrypt}(T,Id_{i}\mathrm{Encrypt}\left(h,T,Id_{i},c\right))=c$.*

**Proof** **of** **Theorem** **1.** According to Equation (8), the encrypted augmented data block $c_{E}$ is given as follows:

(14) $$\begin{array}{cc} c_{E} & =E_{c,T}\cdot \left(p,D\right)\\ \; & =(E_{c,T}\cdot p,E_{c,T}\cdot D), \end{array}$$

Then, according to our scheme, the decryption matrix can be expressed as follows:

(15) $$\begin{array}{cc} c_{D} & =DE_{c,T}\cdot c_{E}\\ \; & =DE_{c,T}\cdot \left(E_{c,T}\cdot p,E_{c,T}\cdot D\right)\\ \; & =\left[\begin{array}{ccc} c_{e}^{-1} & & \\ & \ddots & \\ & & c_{e}^{1} \end{array}\right]\cdot \left(\left[\begin{array}{ccc} c_{e} & & \\ & \ddots & \\ & & c_{e} \end{array}\right]\cdot p,\left[\begin{array}{ccc} c_{e} & & \\ & \ddots & \\ & & c_{e} \end{array}\right]\cdot D\right)\\ \; & =(p,D)\\ \; & =c \end{array}$$

Therefore, The proof is completed. □

**Theorem** **2.** *For any generation $Id_{i}$ and $c\in F{}_{p}^{t+n}$,* ***Verify*** *(pk, c, $Id_{i}$, Δ) is successful.*

**Proof** **of** **Theorem** **2.** According to Equations (9)–(12), we have

(16) $$\begin{array}{cc} \eta_{1} & =e(\Delta ,o)\\ \; & =e(\zeta^{\sum_{i=1}^{t+n}c_{i}sk_{i}+\left(\sum_{i=1}^{t}c_{i}\right)H\left(Id_{i}\right)sk_{t+n+1}},o)\\ \; & =e{\left(\zeta ,o\right)}^{\sum_{i=1}^{t+n}c_{i}sk_{i}+\left(\sum_{i=1}^{t}c_{i}\right)H\left(Id_{i}\right)sk_{t+n+1}} \end{array}$$

and

(17) $$\begin{array}{cc} \eta_{2} & =e(\zeta ,\prod_{i=1}^{t+n}h_{i}^{c_{i}}\cdot \prod_{i-1}^{t}h_{t+n+1}^{H\left(Id_{i}\right)c_{i}})\\ \; & =e(\zeta ,\prod_{i=1}^{t+n}o^{sk_{i}c_{i}}\cdot \prod_{i-1}^{t}o^{sk_{t+n+1}H\left(Id_{i}\right)c_{i}})\\ \; & =e(\zeta ,o^{\sum_{i=1}^{t+n}c_{i}sk+\sum_{i=1}^{t}c_{i}H\left(Id_{i}\right)sk_{t+n+1}})\\ \; & =e{\left(\zeta ,o\right)}^{\sum_{i=1}^{t+n}c_{i}sk_{i}+\left(\sum_{i=1}^{t}c_{i}\right)H\left(Id_{i}\right)sk_{t+n+1}} \end{array}$$

Therefore, $\eta_{1}=\eta_{2}$ can be held for any generation $Id_{i}$ and $c\in F{}_{p}^{t+n}$. □

The security of our privacy-preserving scheme relies on the hardness of the discrete logarithm over G, where for any $x\in Z_{p}^{*}$ and given $\left(g,g^{x}\right)$, *x* cannot be computed in any polynomial algorithm [36].

### 4.3. The Optimization Cluster Formulation Scheme Based on Repeated Game Model

After considering the data privacy and the trustworthiness of IoT devices, an effective trust cluster formulation scheme (ETCFS) is designed based on the repeated game for preserving the network stability and conserving the power consumption due to packet re-transmission. Many studies in the literature have reported that the repeated game model can solve the balance problem between network performance and resource consumption.

#### 4.3.1. Repeated Game Model

In this sub-subsection, we first present a repeated game model based on the trustworthiness to elect the trust route IoT devices. Then, the subgame-perfect Nash equilibrium is given. Furthermore, the repeated game model is formally defined as follows:

(a) Attackers $A_{r}=\left\{A_{1},A_{2},\ldots ,A_{r}\right\}$ and defenders $D_{r}=\left\{D_{1},D_{2},\ldots ,D_{r}\right\}$ are the cooperating parties in the repeated game, where $r\in N_{+}$.
(b) Given the utility function $U_{A}^{r}$ and $U_{D}^{r}$, and the loss discount δ, the average utility is $\lim_{r\to \infty }\sum_{j=1}^{r}\frac{U_{A}}{r}$ and $\lim_{r\to \infty }\sum_{j=1}^{r}\frac{U_{D}}{r}$, where *r* is number of iterations according to the lifetime of the network. Furthermore, the total payoff for both parties are respectively as follows:
  (18) $$U_{A}=U_{A}^{1}+\delta U_{A}^{2}+\delta^{2}U_{A}^{3}+\ldots +\delta^{r-1}U_{A}^{r}=\sum_{r=1}^{r}\delta^{r-1}U_{A}^{r},$$
  (19) $$U_{D}=U_{D}^{1}+\delta U_{D}^{2}+\delta^{2}U_{D}^{3}+\ldots +\delta^{r-1}U_{D}^{r}=\sum_{r=1}^{r}\delta^{r-1}U_{D}^{r},$$
  where the weight of the current and future payoff is inconsistent, and the future payoff is generally less than the weight of the current payoff.
(c) The proposed repeated game model is finite due to the power of the entire network being predetermined. Therefore, the finite repeated game can be solved by the backward method, which basically converges to the sub-game equilibrium.

#### 4.3.2. The Solution of Repeated Game Model for Optimizing Cluster Formulation

In the IoT, the various IoT devices including pads, phones, and monitors are members of the cluster (CM). The network controller hopes that the IoT devices with higher energy and trustworthiness become cluster heads (CH). Furthermore, the energy level *E* of IoT devices is divided into two subsets, that is, $E_{h}$ and $E_{l}$ based on the remaining energy, where $E_{h}$ is the set of nodes having energy more than or equal to the threshold $E_{th}$, and $E_{l}$ is lower than $E_{th}$. Each IoT device in the cluster can select CH or CM according to the two strategies S=CH,CM. In addition, the payoff of players can be found in Table 1.

Meanwhile, the network controller is a defender, and other IoT devices may be normal or malicious, so the utility function $U_{A}^{r}$ and $U_{D}^{r}$ in the iteration *r* can be defined as follows:

(a) Suppose that all members of $E_{h}$ and $E_{l}$ IoT devices become CH with no CM, and the payoffs of defender and attacker are decreasing. At this time, the cluster is illegal. Therefore, the utility of defender and attacker in the iteration *r* can be expressed as
  (20) $$\left\{\begin{array}{c} U_{D,T}^{r}=\alpha T_{h}-2\theta C_{h}\\ U_{A,T}^{r}=\alpha T_{l}, \end{array}\right.$$
  where α and θ are the weights of the reward and penalty, α+θ=1, α,θ∈[0,1]. $T_{h}$ and $T_{l}$ are the trustworthiness of low-energy and high-energy IoT devices. Meanwhile, $C_{h}$ and $C_{l}$ are the communication costs of high-energy and low-energy IoT devices.
(b) Suppose that $E_{h}$ and $E_{l}$ IoT devices respectively become CH and CM; the payoff of the defender is the highest, and that of the attacker is the lowest. Therefore, the utility of the defender and attacker in the iteration *r* can be expressed as
  (21) $$\left\{\begin{array}{c} U_{D,T}^{r}=2\alpha T_{h}-\theta C_{h}\\ U_{A,T}^{r}=\alpha T_{l}-2\theta C_{l}, \end{array}\right.$$
(c) Suppose that $E_{l}$ and $E_{h}$ IoT devices respectively become CH and CM, and the payoffs of the attacker are the highest. However, the CH with $E_{l}$ can also help the network controller formulate a legal cluster. Therefore, the weight of reward and penalty are predefined, and the utility of defender and attacker in the iteration *r* are given by
  (22) $$\left\{\begin{array}{c} U_{D,T}^{r}=T_{h}-\theta C_{h}\\ U_{A,T}^{r}=2\alpha T_{l}-\theta C_{l}, \end{array}\right.$$
(d) Suppose that $E_{l}$ and $E_{h}$ IoT devices have become CM with no CH; then, the cluster is illicit. Therefore, the respective utilities of the defender and attacker in the iteration *r* are given by
  (23) $$\left\{\begin{array}{c} U_{D,T}^{r}=\alpha T_{h}-2\theta C_{h}\\ U_{A,T}^{r}=\alpha T_{l}-\theta C_{l}, \end{array}\right.$$

Then, we achieve subgame-perfect Nash equilibrium $\left(\psi^{*},\xi^{*}\right)=\left(1,0\right)$ according to the evolutionarily stable strategy (ESS) [35]. The details can be seen in Appendix A.

## 5. Simulation Result and Discussion

This section shows the simulation result of the energy-efficient privacy-preserving scheme and ETCFS scheme in IoTs. We use the OMNET++ simulator to construct the network model with malicious activity and compute the trustworthiness of each IoT device. The details of parameters used to configure the network model are given in Table 2. Then, we compare the ETCFS scheme with state-of-the-art TDDG [33], HIDS [37], and LHIDS [38] to show the effectiveness of the above schemes. In addition, the maximum running iteration of the simulation is 100.

### 5.1. Simulation Parameter Setting

In this subsection, we define the metrics, including trustworthiness, the running time of the privacy-preserving scheme, and the lifetime of the IoT, to discuss the performance of the privacy-preserving scheme and ETCFS scheme.

(a) The trustworthiness of each IoT device consists of direct trust $T_{direct}$ and indirect trust $T_{indirect}$. The total trust is defined as follows:
  (24) $$T_{total}=\lambda_{1}T_{direct}+\lambda_{2}T_{indirect}$$
  where $\lambda_{1}$ and $\lambda_{2}$ are the weight parameters of direct and indirect trust, which satisfy $\lambda_{1}+\lambda_{2}=1$. The trust evaluation method including direct and indirect trust can be found in [39].
(b) The running time of the privacy-preserving scheme reflects the effectiveness of our scheme, which can run faster than previous schemes [40,41], while satisfying the demand for data privacy.
(c) The lifetime of the IoT reflects lower resource consumption than in other literature. Furthermore, the lifetime of IoTs with our repeated game model is the highest.

### 5.2. Performance Comparison

In this subsection, we compare the performance of the proposed privacy-preserving and ETCFS scheme with the state-of-the-art methods under the preset network parameters.

**Figure 2:**
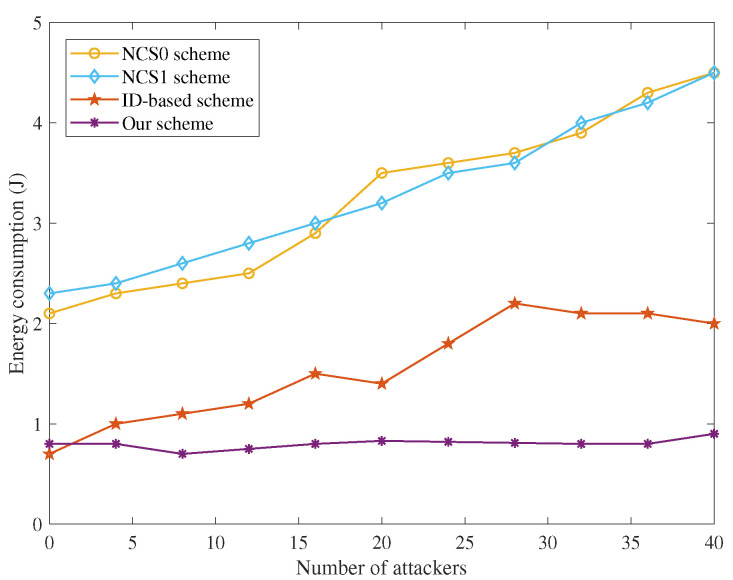
The energy consumption with T-S fuzzy trust model.

**Figure 3:**
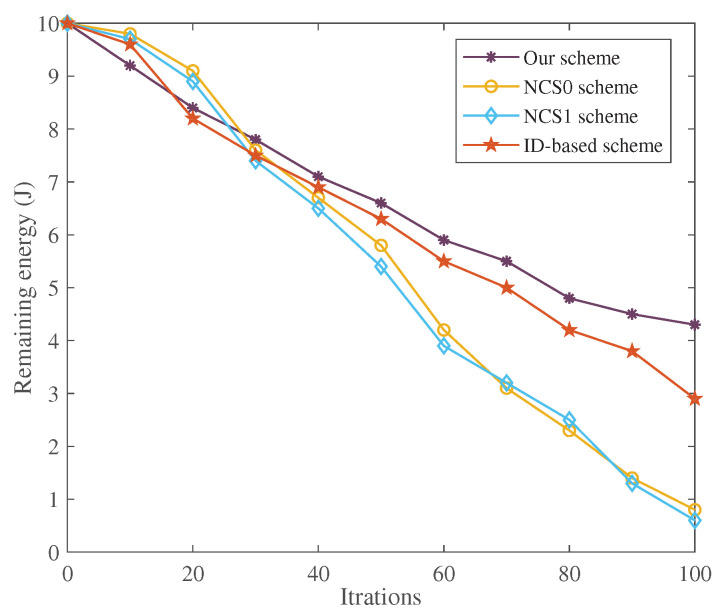
The remaining energy for the T-S fuzzy trust model with different iterations.

**Figure 4:**
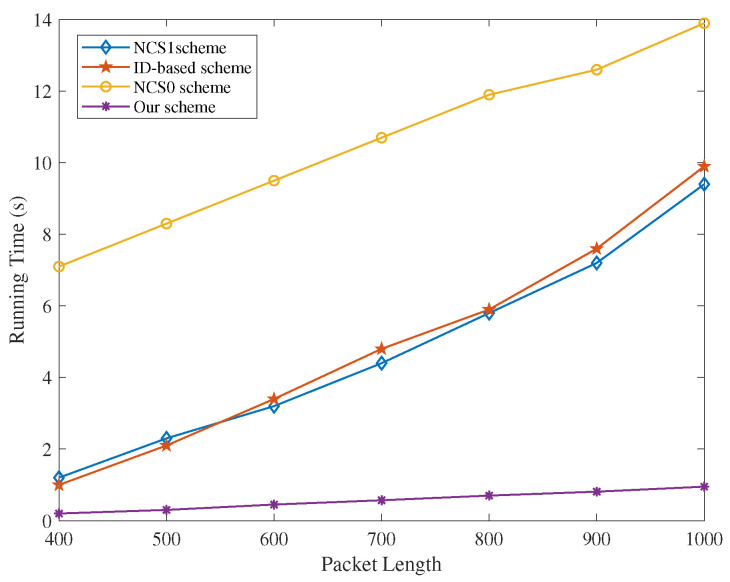
The running time of signature, encryption, and verification in different schemes against packet length.

**Figure 5:**
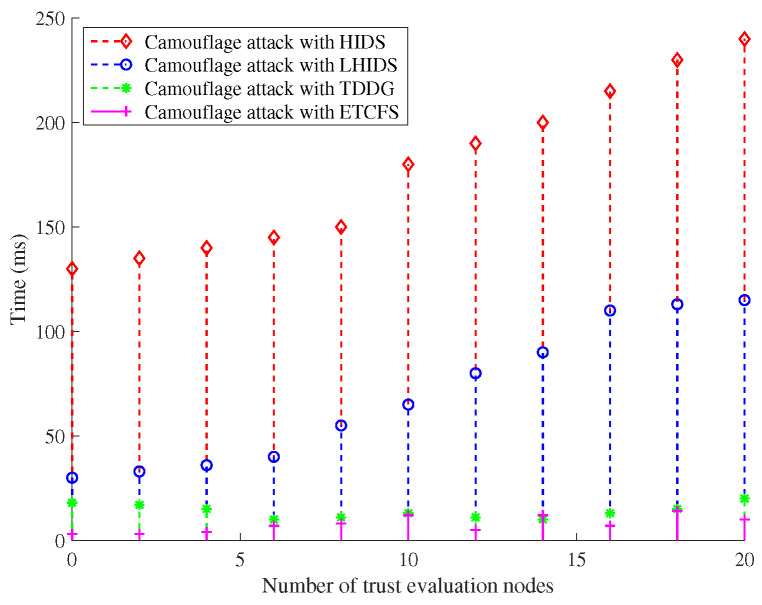
The time consumption with cluster formulation under different schemes in camouflage attack (hop limit = 1).

**Figure 6:**
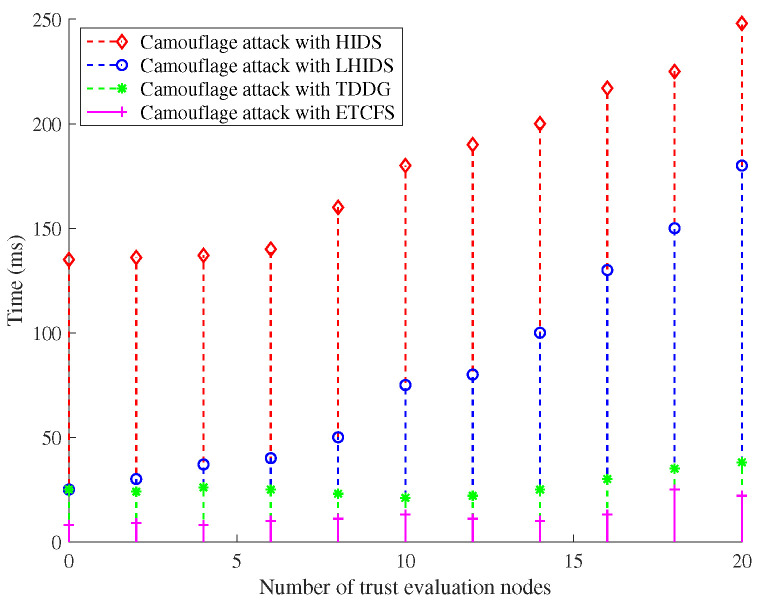
The time consumption with cluster formulation under different schemes in camouflage attack (hop limit = 2).

(a) Energy Efficiency with T-S Fuzzy Trust Model: In Figure 2 and Figure 3, the energy consumption of our T-S fuzzy trust model is compared with NCS0-, NCS1-, and ID-based schemes. The result of the simulation shows that our scheme has the lowest energy consumption. As the number of attack nodes in the IoT increases, the energy required for trust evaluation gradually increases. However, the energy consumption of our scheme has been in a stable state, and there is no significant increase. Meanwhile, our scheme has the highest remaining energy than other schemes when the iteration= [30–100].
(b) Time Efficiency of Our Privacy-Preserving Scheme: In Figure 4, the runtime of our trust-based privacy-preserving scheme is the lowest compared to the other three methods. In addition, our scheme has higher stability according to the magnitude of running time variation.
(c) Time Consumption with Cluster Formulation: In Figure 5 and Figure 6, we compare the time consumption when the hop limit is 1 and 2 under camouflage attack. Based on theoretically verifying that the proposed repeated game has effective game equilibrium, we also find our game-based cluster formulation has the lowest time consumption.

## 6. Conclusions

This paper investigates a novel fuzzy-based privacy-preserving scheme to defend against pollution attacks in coding IoTs and constructs a repeated game model to balance data security and energy consumption. We propose a T-S fuzzy trust evaluation method to replace the traditional cryptography scheme and reduce the energy consumption in IoTs. Then, we introduce the trust-based privacy-preserving scheme, in which the security relies on the hardness of the discrete logarithm. Finally, an optimal cluster formulation based on the repeated game model is proposed to balance the data security and energy consumption. The result shows that the cluster formulation can mitigate the camouflage attack. In addition, our scheme only considers two types of attacks in IoTs. Therefore, we will consider more kinds of attacks on IoT data, and construct more effective privacy-preserving schemes in future work.

## Figures and Tables

**Table 1:** The different payoffs under different behaviors of players.

Strategy	To Be CH	To Be CM
Normal	$U_{i,j}\left(N,CH\right)$, $U_{i,j}^{\prime }\left(N,CH\right)$	$U_{i,j}\left(N,CM\right)$, $U_{i,j}^{\prime }\left(N,CM\right)$
Malicious	$U_{i,j}\left(M,CH\right)$, $U_{i,j}^{\prime }\left(M,CH\right)$	$U_{i,j}\left(M,CM\right)$, $U_{i,j}^{\prime }\left(M,CM\right)$

**Table 2:** The simulation network parameters.

Parameter	Value	Parameter	Value
Network region	200 × 200 m ${}^{2}$	Communication radius	2 m
Number of IoT devices	100	Sensing radius	1 m
Initial trustworthiness	0.6	Attack intensity	0.2–0.6
Packet length	400–1000	α,θ,δ	0.2, 0.2, 0.4
Initial energy	10 J	Maximum iteration	100
*E*th	4 J	Hop limit	2

## Data Availability

Data sharing not applicable.

## Appendix A

### Appendix A.1. The Optimizing Solution of Defenders

(A1) $$\begin{array}{cc} U_{D}^{E_{h}\to \mathrm{CH}} & =\sum_{r=1}^{r}\delta^{r-1}\left[\xi \left(\alpha T_{h}-2\theta C_{h}\right)+\left(1-\xi \right)\left(2\alpha T_{h}-\theta C_{h}\right)\right]\\ U_{D}^{E_{h}\to \mathrm{CM}} & =\sum_{r=1}^{r}\delta^{r-1}\left[\xi \left(T_{h}-\theta C_{h}\right)+\left(1-\xi \right)\left(\alpha T_{h}-2\theta C_{h}\right)\right]\\ U_{D}^{E_{l}\to \mathrm{CH}} & =\sum_{r=1}^{r}\delta^{r-1}\left[\xi \left(\alpha T_{h}-2\theta C_{h}\right)+\left(1-\xi \right)\left(T_{h}-\theta C_{h}\right)\right]\\ U_{D}^{E_{l}\to \mathrm{CM}} & =\sum_{r=1}^{r}\delta^{r-1}\left[\xi \left(2\alpha T_{h}-\theta C_{h}\right)+\left(1-\xi \right)\left(\alpha T_{h}-2\theta C_{h}\right)\right], \end{array}$$

(A2) $$\begin{array}{cc} \bar{U_{D}} & =\psi U_{D}^{E_{h}\to \mathrm{CH}}+\left(1-\psi \right)U_{D}^{E_{h}\to \mathrm{CM}}+\left(1-\psi \right)U_{D}^{E_{l}\to \mathrm{CH}}+\psi U_{D}^{E_{l}\to \mathrm{CM}}\\ \; & =\sum_{r=1}^{r}\delta^{r-1}\left[\begin{array}{c} \psi \xi \left(\alpha T_{h}-2\theta C_{h}\right)+\psi \left(1-\xi \right)\left(2\alpha T_{h}-\theta C_{h}\right)\\ +\left(1-\psi \right)\xi \left(T_{h}-\theta C_{h}\right)+\left(1-\psi \right)\left(1-\xi \right)\left(\alpha T_{h}-2\theta C_{h}\right)\\ +\left(1-\psi \right)\xi \left(\alpha T_{h}-2\theta C_{h}\right)+\left(1-\psi \right)\left(1-\xi \right)\left(T_{h}-\theta C_{h}\right)\\ +\psi \xi \left(2\alpha T_{h}-\theta C_{h}\right)+\psi \left(1-\xi \right)\left(\alpha T_{h}-2\theta C_{h}\right) \end{array}\right]\\ \; & =\sum_{r=1}^{r}\delta^{r-1}\left[\left(\alpha T_{h}-2\theta C_{h}\right)+\left(2\alpha T_{h}-\theta C_{h}\right)\psi +\left(T_{h}-\theta C_{h}\right)\left(1-\psi \right)\right]\\ \; & =\sum_{r=1}^{r}\delta^{r-1}\left[\left(1+2\psi \right)\alpha T_{h}-3\theta C_{h}+T_{h}\left(1-\psi \right)\right] \end{array}$$

(A3) $$\begin{array}{cc} \frac{d\psi }{dr} & =\psi (U_{D}^{E_{h}\to \mathrm{CH}}+U_{D}^{E_{l}\to \mathrm{CM}}-\bar{U_{D}})\\ \; & =\psi \sum_{r=1}^{r}\delta^{r-1}\left[\begin{array}{c} \xi \left(\alpha T_{h}-2\theta C_{h}\right)+\left(1-\xi \right)\left(2\alpha T_{h}-\theta C_{h}\right)\\ +\xi \left(2\alpha T_{h}-\theta C_{h}\right)+\left(1-\xi \right)\left(\alpha T_{h}-2\theta C_{h}\right)\\ -[\left(1+2\psi \right)\alpha T_{h}-3\theta C_{h}+T_{h}\left(1-\psi \right)] \end{array}\right]\\ \; & =\psi \sum_{r=1}^{r}\delta^{r-1}\left[\left(2-2\psi \right)\alpha T_{h}-\left(1-\psi \right)T_{h}\right)]\\ \; & =\sum_{r=1}^{r}\delta^{r-1}\psi \left(1-\psi \right)\left(2\alpha T_{h}-T_{h}\right) \end{array}$$

when $\frac{d\psi }{dr}=0$, the player *D* achieves a stable state. Therefore, when ψ*=0 or 1, the player *D* has the highest payoff.

### Appendix A.2. The Optimizing Solution of Attackers

Similarly, the expression of player *A* is obtained as follows:

(A4) $$\begin{array}{cc} U_{A}^{E_{h}\to \mathrm{CH}} & =\sum_{r=1}^{r}\delta^{r-1}\left[\psi \left(\alpha T_{l}\right)+\left(1-\psi \right)\left(\alpha T_{l}-2\theta C_{l}\right)\right]\\ U_{A}^{E_{h}\to \mathrm{CM}} & =\sum_{r=1}^{r}\delta^{r-1}\left[\psi \left(2\alpha T_{l}-\theta C_{l}\right)+\left(1-\psi \right)\left(\alpha T_{l}-\theta C_{l}\right)\right]\\ U_{A}^{E_{l}\to \mathrm{CH}} & =\sum_{r=1}^{r}\delta^{r-1}\left[\psi \left(\alpha T_{l}\right)+\left(1-\psi \right)\left(2\alpha T_{l}-\theta C_{l}\right)\right]\\ U_{A}^{E_{l}\to \mathrm{CM}} & =\sum_{r=1}^{r}\delta^{r-1}\left[\psi \left(\alpha T_{l}-2\theta C_{l}\right)+\left(1-\psi \right)\left(\alpha T_{l}-\theta C_{l}\right)\right] \end{array}$$

(A5) $$\begin{array}{cc} \bar{U_{A}} & =\xi U_{A}^{E_{h}\to \mathrm{CH}}+\left(1-\xi \right)U_{A}^{E_{h}\to \mathrm{CM}}+\left(1-\xi \right)U_{A}^{E_{l}\to \mathrm{CH}}+\xi U_{A}^{E_{l}\to \mathrm{CM}}\\ \; & =\sum_{r=1}^{r}\delta^{r-1}\left[\begin{array}{c} \xi \left(\alpha T_{l}-2\theta C_{l}\right)+\left(1-\xi \right)\left(2\alpha T_{l}-\theta C_{l}\right)\\ +\left(1-\psi \right)\left(\alpha T_{l}-\theta C_{l}\right)+\psi \left(\alpha T_{l}\right) \end{array}\right]\\ \; & =\sum_{r=1}^{r}\delta^{r-1}\left[\left(3-\xi \right)\alpha T_{l}+\left(\psi -\xi -2\right)\theta C_{l}\right] \end{array}$$

(A6) $$\begin{array}{cc} \frac{d\xi }{dr} & =\sum_{r=1}^{r}\delta^{r-1}\xi \left(U_{A}^{E_{h}\to \mathrm{CM}}+U_{A}^{E_{l}\to \mathrm{CH}}-\bar{U_{A}}\right)\\ \; & =\sum_{r=1}^{r}\delta^{r-1}\xi \left[\begin{array}{c} \psi \left(2\alpha T_{l}-\theta C_{l}\right)+\left(1-\psi \right)\left(\alpha T_{l}-\theta C_{l}\right)+\psi \left(\alpha T_{l}\right)\\ +\left(1-\psi \right)\left(2\alpha T_{l}-\theta C_{l}\right)-\left(3-\xi \right)\alpha T_{l}-\left(\psi -\xi -2\right)\theta C_{l} \end{array}\right]\\ \; & =\sum_{r=1}^{r}\delta^{r-1}\xi \left[\left(\psi +\xi \right)\alpha T_{l}+\xi \theta C_{l}\right] \end{array}$$

When the $\psi^{*}=0$; the $\xi^{*}=0$, relatively; the $\psi^{*}=1$; the $\xi^{*}=0$; and the optimizing payoffs of defender and attacker can be achieved. Furthermore, according to the reality, the subgame-perfect nash equilibrium is obtained as $\left(\psi^{*},\xi^{*}\right)=\left(1,0\right)$.
